# HyperACP: A cutting-edge hybrid framework for anticancer peptide classification via scalable feature extraction and adaptive neighbor-based synthesis

**DOI:** 10.1371/journal.pcbi.1013489

**Published:** 2025-09-11

**Authors:** Bangyi Zhang, Yun Zuo, Jun Wan, Jiayue Liu, Xiangrong Liu, Xiangxiang Zeng, Zhaohong Deng

**Affiliations:** 1 School of Artificial Intelligence and Computer Science, Jiangnan University, Wuxi, China; 2 Department of Computer Science and Technology, National Institute for Data Science in Health and Medicine, Xiamen Key Laboratory of Intelligent Storage and Computing, Xiamen University, Xiamen, China; 3 School of Information Science and Engineering, Hunan University, Yuelu, Changsha, China; Xinjiang Technical Institute of Physics and Chemistry, CHINA

## Abstract

Cancer remains a major contributor to global mortality, constituting a significant and escalating threat to human health. Anticancer peptides (ACPs) have emerged as promising therapeutic agents due to their specific mechanisms of action, pronounced tumor-targeting capability, and low toxicity. Nevertheless, traditional approaches for ACP identification are constrained by their reliance on shallow, hand-crafted sequence features, which fail to capture deeper semantic and structural characteristics. Moreover, such models exhibit limited robustness and interpretability when confronted with practical challenges such as severe class imbalance. To address these limitations, this study proposes HyperACP, an innovative framework for ACP recognition that integrates deep representation learning, adaptive sampling, and mechanistic interpretability. The framework leverages the ESMC protein language model to extract comprehensive sequence features and employs a novel adaptive algorithm, ANBS, to mitigate class imbalance at the decision boundary. For enhanced model transparency, SHAP-Res is incorporated to elucidate the contributions of individual residues to the final predictions. Comprehensive evaluations demonstrate that HyperACP consistently outperforms state-of-the-art methods across multiple datasets and validation protocols—including 10-fold cross-validation and independent test sets—according to metrics such as Accuracy (ACC), Sensitivity (SN), Specificity (SP), Matthews Correlation Coefficient (MCC), and Area Under the Curve (AUC). Furthermore, the model yields biologically interpretable results, pinpointing key residues (K, L, F, G) known to play pivotal roles in anticancer activity. These findings provide not only a robust predictive tool (available at www.hyperacp.com) but also novel insights into the structure-function relationships underlying ACPs.

## Author summary

Accurately identifying anticancer peptides (ACPs) is crucial for the discovery of next-generation cancer therapeutics, but existing computational methods often struggle with feature limitations, data imbalance, and poor interpretability. In this study, we present HyperACP—a novel computational framework that combines advanced deep protein representation, an adaptive sampling algorithm, and an ensemble learning strategy to systematically address these challenges. HyperACP utilizes a cutting-edge protein language model to capture the complex structural and functional information embedded in peptide sequences. Our newly designed sampling method generates informative minority class samples in challenging boundary regions, improving the model’s robustness to imbalanced data. Most importantly, we introduce SHAP-Res, a residue-level interpretability module that reveals how individual amino acids drive model predictions and links computational insights directly to biological function. HyperACP not only achieves state-of-the-art prediction accuracy but also provides transparent and biologically meaningful explanations, paving the way for more reliable and interpretable peptide-based drug discovery.

## Introduction

Cancer remains a critical global public health challenge, as conventional treatments often fail to meet diverse clinical needs [1,2]. This highlights the urgent demand for novel therapeutic strategies that are mechanistically defined, highly specific, and minimally toxic [3]. In this context, ACPs, short-chain peptides with potent and selective antitumor activity, have emerged as a promising solution. Their mechanisms, including membrane disruption and apoptosis induction, grant them significant advantages over conventional chemotherapy, such as low toxicity and a reduced risk of drug resistance [4]. However, identifying and designing ACPs is hampered by their reliance on complex sequence-structure relationships, limited data, and high experimental costs [5]. These challenges underscore the necessity for high-performance computational models to accelerate the discovery and development of ACP-based anticancer therapies [6,7].

Recent advances in artificial intelligence have spurred the development of numerous computational methods for ACP prediction [8–12]. Early approaches often relied on traditional machine learning with sophisticated feature engineering. For instance, ACPred uses sequence-derived features with a Support Vector Machine (SVM), while mACPpred integrates multiple encoding schemes through a two-stage feature selection pipeline, achieving performance superior to single-feature models [13–15]. Other advanced models like ACP-ML employ ensemble learning strategies with multi-stage feature filtering, yielding high accuracy on independent test sets [16]. More recently, deep learning models have been introduced to automatically capture sequence-order information [17]. Methods like ACPred-BMF and iDACP utilize Bi-LSTMs to learn long-range dependencies within peptide sequences [14,18]. Despite these advancements, several critical limitations persist. First, most methods depend heavily on handcrafted feature encodings (e.g., AAC, DPC, CTD), restricting their ability to learn global semantic information and recognize diverse peptides [19–21]. Second, the small scale of public ACP datasets (typically hundreds to a few thousand sequences) constrains the generalization power of deep learning models [22,23]. Finally, even with interpretability tools like attention mechanisms or SHAP, most deep learning models remain black boxes, offering limited insight into the biological basis of their predictions.

**Fig 1:**
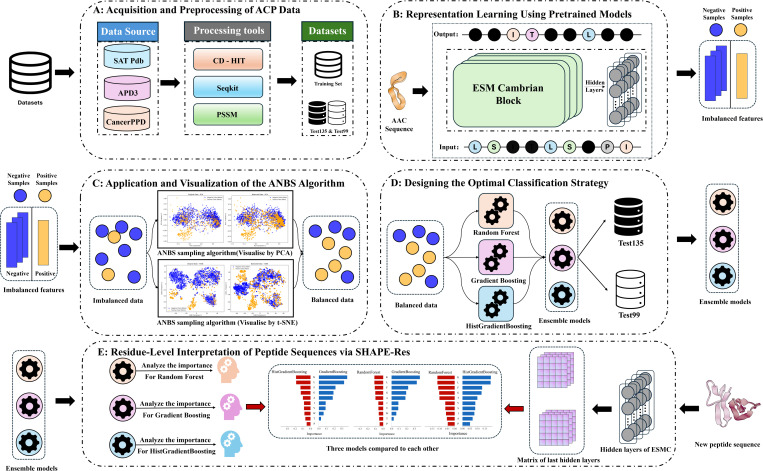
Framework of HyperACP.

To address the aforementioned challenges, we introduce HyperACP, a novel framework for enhancing ACP prediction accuracy, robustness, and biological credibility. The process begins with constructing a high-quality dataset through rigorous preprocessing (Fig 1A), after which sequences are encoded by ESMC model into high-dimensional embeddings that capture both contextual and structural properties (Fig 1B) [22,24–27]. To overcome the constraints of small dataset sizes and severe class imbalance, we propose the Adaptive Neighborhood-Based Sampling (ANBS) algorithm. This method identifies majority-class samples near the minority class and synthesizes new samples via interpolation, improving local class separability while preserving the global data structure (Fig 1C-D). The resulting balanced representations are used to train a robust ensemble of classifiers [28–30]. For interpretability, we developed SHAP-Res, a novel residue-level explanation method that aligns global SHAP attributions with ESMC’s residue embeddings (Fig 1E). This mapping provides a biologically grounded attribution of model decisions to individual amino acids, uncovering functional patterns. Crucially, SHAP-Res represents the first semantic-to-structure mapping between language model embeddings and residue-level biological relevance in this field.

We conducted comprehensive evaluations to demonstrate the superiority of our model, HyperACP. Benchmark comparisons show that HyperACP achieves new state-of-the-art performance, outperforming existing tools across various metrics. Furthermore, rigorous ablation studies validate the distinct contributions of our proposed ANBS sampling strategy and ESMC representation model, confirming their individual effectiveness.

## Materials and methods

### Benchmark dataset

The benchmark dataset for this study was compiled from three public databases: CancerPP, APD3, and SATPdb, initially comprising 1,350 experimentally validated ACP sequences. To mitigate homology bias, redundant sequences with more than 90% similarity were removed using the CD-HIT tool at a 90% identity threshold. Subsequently, sequences were filtered using Seqkit to retain only those with lengths between 5 and 50 amino acid residues, a range characteristic of most functional peptides. A final filtering step retained only sequences for which a Position-Specific Scoring Matrix (PSSM) could be generated via PSI-BLAST, resulting in a curated set of 622 ACP samples. Negative samples were randomly selected from peptides with no known anticancer activity. These were subjected to an identical processing pipeline, including the same homology reduction and length-filtering criteria, followed by the extraction of corresponding PSSM features. This process yielded 1,839 non-ACP samples. The final training set was constructed by randomly sampling 487 positive and 1,479 negative samples from these curated sets.

To evaluate the model’s generalization ability, two independent test sets were also introduced: ACP135, comprising 135 positive and 360 negative samples derived from the portions of the initial datasets not used in training, and ACP99, a dataset constructed by Agrawal et al. containing 99 positive and 157 negative samples. Both test sets underwent the same processing workflow, including homology removal and PSSM feature extraction. Detailed statistics of all datasets are provided in Table 1.

**Table 1:** Dataset for ACPs.

Name	Negative samples	Positive samples
**Training set**	1479	487
**Test135**	360	135
**Test99**	157	99

### Feature extraction method based on ESM Cambrian

ESMC is a member of the ESM3 family, representing a state-of-the-art protein language model specifically developed to advance protein representation learning [27]. By leveraging unsupervised learning on large-scale protein sequence data, ESMC is capable of automatically extracting deep biological features, thereby eliminating the dependence on labeled data and manual feature engineering inherent in traditional approaches. In contrast to ESM3, which emphasizes controllable protein generation, ESMC is primarily oriented toward capturing the intrinsic biological representations of proteins. Designed as the successor to ESM2, ESMC offers notable enhancements in both predictive performance and computational efficiency. ESMC utilizes a generative masked language model (MLM) objective for training, where the training objective is formulated as the negative log-likelihood (NLL):


$$L=-{\text{E}}_{x,m}[\frac{\text{1}}{\left|m\right|}\sum_{i\in m}\text{log}p(x_{i}|x_{-m})]$$ (1)

In this equation, x  denotes the protein sequence, m refers to the set of masked positions, and ${x\;}_{-m}$ represents the sequence with the masked positions excluded. The model is thus trained by masking a subset of amino acids within the sequence and predicting the identities of these masked residues, thereby enabling the extraction of rich sequence representations. Through iterative training, the model effectively learns to generate protein sequences and predict their structural and functional properties. Furthermore, structural prediction can be incorporated using a similar generative strategy, wherein the three-dimensional structure of the protein is discretized into structural tokens that are subsequently processed by the model:


$$\hat{S}=\text{Decoder}(\hat{X})$$ (2)

In this equation, $\hat{X}$ denotes the sequence representation generated by the model, while $\hat{S}$ corresponds to the final predicted protein structure. By jointly training on both sequence and structural tokens, ESM3 is capable of directly generating the three-dimensional structure of a protein, thereby eliminating the need for a separate structure prediction module [26].

Compared to traditional large-scale models, ESMC achieves performance comparable to larger models (e.g., 650M parameters) with significantly fewer parameters (e.g., 300M and 600M), while also providing faster inference and greater computational efficiency. For example, the 300M-parameter version of ESMC delivers predictive accuracy similar to the 650M-parameter ESM2, but with reduced memory requirements. Notably, the 6B-parameter ESMC demonstrates substantial improvements in performance, reflecting excellent scalability and efficiency. These characteristics confer clear advantages to ESMC in protein representation learning and downstream applications.

In contrast to conventional amino acid sequence feature extraction methods—such as amino acid composition (AAC), dipeptide composition (DC), and pseudo-amino acid composition (PAAC)—which primarily extract single or combined frequency features and physicochemical properties using manually defined rules [31], ESMC employs a deep unsupervised learning approach to automatically learn functional and structural protein representations from large-scale datasets. The feature dimensionality is determined by the model’s parameter scale, enabling the extraction of richer biological information without the need for manual labeling or feature engineering. This capability is particularly advantageous in scenarios lacking explicit structural or functional annotations. Table 2 summarizes the feature dimensionalities and key characteristics of different feature extraction methods.

**Table 2:** Comparison of amino acid sequence feature extraction methods.

Methods	Dim	Description
**AAC**	20	Frequency of 20 amino acids
**DC**	400	Frequency of dipeptides
**PAAC**	20 + N	AAC + additional features
**Kmer**	Variable	Frequency of k-mers
**CTD**	60	Composition, transition, distribution
**TDC**	64	Frequency of trinucleotides
**SOCN**	400	Sequence order coupling of dipeptides
**AC**	20	Amino acid physicochemical properties
**ESMC(300M)**	960	Deep learning, large-scale, unsupervised
**ESMC(600M)**	1152	
**ESMC(6B)**	2560	

Traditional protein feature extraction methods frequently suffer from redundancy and limited expressiveness. For instance, both AAC and PAAC predominantly capture amino acid frequency information, resulting in repetitive features, while methods such as DC and TDC, despite incorporating combinations of amino acids, are insufficient in capturing higher-order structural patterns within protein sequences and thus offer a restricted feature scope. In contrast, ESMC leverages deep unsupervised learning to automatically extract complex, high-dimensional features from large-scale datasets. This approach not only addresses the redundancy inherent in traditional feature concatenation but also enables a more comprehensive and in-depth learning of protein structural and functional characteristics, thereby conferring superior representational power and generalization capability.

### ADASYN algorithm

In studies aimed at addressing class imbalance, the Adaptive Synthetic Sampling (ADASYN) algorithm is frequently employed to augment minority class samples and enhance the model’s capacity to learn from underrepresented categories. Unlike traditional oversampling approaches such as SMOTE, ADASYN introduces an adaptive mechanism that assigns varying degrees of emphasis to samples based on their relative difficulty within the feature space. The algorithm selectively generates new minority class samples by considering the local distribution of majority class samples surrounding each minority instance [22,23]. This adaptive strategy not only increases the diversity of the minority class but also mitigates the risk of producing redundant or less informative samples, thereby improving the model’s discriminative ability, particularly near class boundaries and in the presence of outliers.

Specifically, ADASYN quantifies the difficulty associated with each minority class sample $x_{i}$ by evaluating the proportion of majority class samples among its k nearest neighbors:


$$r_{i}=\frac{\Delta_{i}}{k}$$ (3)

In this formulation, $\Delta_{i}$ denotes the number of majority class samples among the k-nearest neighbors of $x_{i}$, while $r_{i}\in [\text{0},\text{1}]$ quantifies the difficulty level associated with each sample. A larger value of $r_{i}$, indicates that the sample resides in a more complex or challenging region of the feature space, thereby requiring the generation of more synthetic samples. All $r_{i}$ values are then normalized as follows:


$$\tilde{r_{\dot{i}}}=\frac{r_{i}}{\sum_{j=\text{1}}^{n}r_{j}}$$ (4)

Accordingly, the number of synthetic samples to be generated for each minority class instance is determined as follows:


$$Gi=\tilde{r_{\dot{i}}}\cdot G$$ (5)

In this context, G denotes the total number of synthetic samples to be generated. For each $x_{i}$, synthetic samples are created by randomly performing linear interpolation among its k nearest minority class neighbors:


$$x_{new}=x_{i}+\delta \cdot (x_{zi}-x_{i}),\delta \sim u(\text{0},\text{1})$$ (6)

This process leads to the generation of a greater number of synthetic samples in regions near the decision boundary, thereby enhancing the model’s capacity to learn from challenging and ambiguous areas within the feature space.

### ANBS algorithm

To address the sample imbalance observed in this study, a neighborhood selection and adaptive synthesis-based sampling algorithm, termed ANBS, is proposed. ANBS iteratively augments the minority class to achieve class balance by selectively generating new samples. The algorithm operates by computing the Euclidean distances between majority and minority class samples and, in each iteration, identifies the minority class sample closest to the majority class. New synthetic samples are then generated for these minority class instances, progressively increasing the representation of the minority class. For two points in the feature space, $p\text{1}=(p_{\text{1}x},p_{\text{1}y},\ldots ,p_{\text{1}n})$ and $p\text{2}=(p_{\text{2}x},p_{\text{2}y},\ldots ,p_{\text{2}n})$, the Euclidean distance is defined as follows:


$$d\left(p_{\text{1}i},p_{\text{2}i}\right)=\sqrt{\sum_{i=\text{1}}^{n}{{(p}_{\text{1}i}-p_{\text{2}i})}^{\text{2}}}\;$$ (7)

Traditional oversampling methods, such as SMOTE, are commonly employed to address class imbalance; however, they often rely on simplistic neighborhood sampling or direct duplication of minority class instances [22]. These approaches may lead to overfitting or introduce artifacts in the data distribution, especially when applied to complex biological datasets. The ANBS algorithm builds upon the principles of ADASYN, adaptively generating synthetic samples in accordance with the distributional boundaries of minority instances. By focusing on boundary regions and enhancing diversity among newly generated samples, ANBS mitigates the risk of redundancy and improves data representativeness. Additionally, the algorithm incorporates a flexible class balancing mechanism via a user-defined target ratio (max_ratio), ensuring that the final sample distribution is aligned with practical task requirements. Compared with conventional oversampling techniques, ANBS provides better control over data heterogeneity and promotes improved model generalization, making it well-suited for classification problems involving limited and highly imbalanced datasets. The pseudocode for ANBS is presented below:

**Algorithm 1. ANBS.**

**Input:**

    $\begin{array}{c} \;D_{maj} \end{array}$, $\begin{array}{c} D_{min} \end{array}$, $\begin{array}{c} {ratio}_{max} \end{array}$, $\begin{array}{c} {iter}_{max} \end{array}$

**Output:**

    $\begin{array}{c} {\;D}_{maj}^{*},{\;D}_{min}^{*} \end{array}$

1: **for** $\begin{array}{c} t\;=\;1 \end{array}$ to $\begin{array}{c} {iter}_{max} \end{array}$ **do**

2:  $\begin{array}{c} K\leftarrow |D_{min}| \end{array}$

3:  $\begin{array}{c} D_{temp}\leftarrow Boundary(D_{maj},\;D_{min},\;K) \end{array}$

4:  $\begin{array}{c} D_{maj}\leftarrow D_{maj}\backslash D_{temp} \end{array}$

5:  $\begin{array}{c} D_{min}\leftarrow D_{min}\cup Synthesize(D_{min},\;K) \end{array}$

6:  if $\begin{array}{c} |D_{min}|\;/\;|D_{maj}|\;\geq \;{ratio}_{max} \end{array}$

     then break

7: end for

8: **Return** $\begin{array}{c} D_{maj}^{*},{\;D}_{min}^{*} \end{array}$

As detailed in Algorithm 1, the ANBS algorithm is designed to dynamically balance the class distribution through an iterative process. This is achieved by adaptively augmenting the minority class while concurrently pruning majority class samples situated near the decision boundary. The algorithm comprises three core steps: (1) Identification of Boundary Majority Samples (Line 3): This step identifies majority class instances in the boundary region by calculating their Euclidean distances to all minority class samples. (2) Majority Set Update (Line 4): The boundary samples identified in the previous step are subsequently removed from the majority class set. (3) Minority Sample Synthesis (Line 5): New minority samples are generated using a modified ADASYN method, synthesized based on representative minority samples located near the boundary. Following each iteration, the algorithm checks if the current class ratio has reached a predefined threshold (Line 6). If the condition is met, the iterative process terminates early.

Let N and P denote the initial counts of the majority and minority class samples, respectively, each with dimensionality d. Let T be the maximum number of iterations. Within each iteration *t*, the time complexity is dominated by two primary operations. The first is the computation of Euclidean distances between all current majority samples ($N_{t}$) and minority samples ($P_{t}$), which has a *t*ime complexity of $O(N_{t}\cdot P_{t}\cdot d)$. The second operation is the sorting of the resulting distances, with a complexity of $O(P_{t}\text{log}P_{t})$. Consequently, the combined time complexity per iteration is:


$$O(N_{t}\cdot P_{t}\cdot (d+\text{log}P_{t}))$$ (8)

Additionally, the identified critical minority samples are oversampled using ADASYN. The worst-case time complexity of this step, driven by its neighborhood-based synthesis, is $O((N_{t}+\left|Q_{t}\right|{)}^{\text{2}}\cdot d)$, where $\left|Q_{t}\right|$ is the number of critical samples ($\left|Q_{t}\right|\ll N_{t}$). Thus, the overall worst-case time complexity of the entire algorithm is:


O(T·N·P·(d+logP)) (9)

In practical applications, the sample dimensionality (d) and the number of iterations (T) are typically treated as fixed constants. Furthermore, the total number of samples tends to converge or decrease with each iteration. Consequently, the algorithm exhibits excellent scalability and practical efficiency, particularly for small to medium-sized datasets.

### Evaluation metrics

In this study, five widely used and representative binary classification metrics were employed to comprehensively evaluate the performance of various machine learning models in the ACP classification task. These metrics include ACC, SN, SP, MCC, and AUC. Collectively, they assess not only the overall predictive performance of the models, but also their ability to maintain balance between positive and negative classes, distinguish decision boundaries, and demonstrate robustness under conditions of class imbalance [32–37]. As such, this evaluation system is broadly adopted in binary classification research.


$$ACC=\frac{TP+TN}{TP+FP+TN+FN}$$ (10)

$$SN=\frac{TP}{TP+FN}$$ (11)

$$SP=\frac{TN}{TN+FP}$$ (12)

$$MCC=\frac{TP\times TN-FP\times FN}{\sqrt{\left(TP+FP)\times (TP+FN)\times (TN+FP)\times (TN+FN\right)}}$$ (13)

$$AUC=\sum_{i=\text{1}}^{n-\text{1}}\left(\frac{FPR_{i+\text{1}}-FPR_{i}}{\text{2}}\times (TPR_{i+\text{1}}+TPR_{i})\right)$$ (14)

$$F\text{1 }Score=\text{2}*\frac{Precision*Recall}{Precision+Recall},\;Precision=\;\frac{TP}{TP+FP}$$ (15)

In this context, TP (True Positive) denotes the number of positive samples correctly identified by the model, while TN (True Negative) represents the number of negative samples correctly classified. FP (False Positive) refers to negative samples incorrectly predicted as positive, and FN (False Negative) refers to positive samples incorrectly classified as negative. Based on these four fundamental quantities, several key evaluation metrics are derived. Sensitivity (or Recall) reflects the proportion of actual positive samples correctly identified and is indicative of the model’s ability to minimize missed detections. Specificity measures the proportion of actual negative samples correctly recognized, thereby controlling the false positive rate and contributing to model stability. Accuracy quantifies the overall correctness of predictions. The MCC, which incorporates all four outcomes (TP, TN, FP, FN), is particularly robust in scenarios with imbalanced class distributions and is widely regarded as the gold standard for binary classification performance. The AUC assesses the model’s discrimination capability across all threshold settings, reflecting both stability and generalization performance. Collectively, this evaluation system provides a comprehensive, multidimensional assessment of model performance, serving as a solid foundation for the selection and optimization of classification models and sampling strategies in this study.

### SHAP-res: Semantic–attention–enhanced residue-level interpretation

To address the interpretability challenges of complex models, our approach draws upon the principles of SHAP (SHapley Additive exPlanations). SHAP is a widely-used post-hoc explanation framework derived from cooperative game theory and the Shapley value. It quantifies the importance of each feature by calculating its average marginal contribution to a model’s prediction across all possible feature combinations. A primary advantage of SHAP is its ability to provide both local explanations for individual predictions and global insights into overall model behavior, all while maintaining theoretical guarantees of fairness and consistency in its attributions.

This study introduces SHAP-Res, a residue-level interpretability framework designed to elucidate how pretrained protein language models capture structural and functional information within peptide sequences. In contrast to traditional methods that focus on global feature importance (such as SHAP) or rely solely on unanchored attention weights, SHAP-Res integrates contextual embeddings from the ESMC model with SHAP-based attribution scores, thereby enabling a quantitative assessment of each residue’s contribution to model predictions.

Let L denote the length of a peptide sequence, and let the residue embedding matrix generated by the ESMC model be represented as:


$$E={\left[e_{\text{1}},e_{\text{2}},\ldots ,e_{L}\right]}^{⊤}\in R^{L\times d}$$ (16)

where $e_{i}\in R^{d}$ denotes the semantic embedding vector corresponding to the i−th residue, with d representing the embedding dimensionality. The feature importance scores assigned by the classifier, as computed using SHAP, are represented by a weight vector:


$$w_{SHAP}\in R^{d}$$ (17)

The interpretability score for the i−th residue is computed as the inner product of its embedding vector and the corresponding SHAP-derived weight vector:


$$s_{i}=e_{i}\cdot w_{SHAP}$$ (18)

Finally, the residue-level contribution vector is obtained as follows:


$$S={\left[s_{\text{1}},s_{\text{2}},\ldots ,s_{L}\right]}^{⊤}\in R^{L}$$ (19)

For the purposes of visualization and comparative analysis, min–max normalization is applied:


$${\tilde{s}}_{i}=\frac{s_{i}-\text{min}(\text{s})}{\text{max}(\text{s})-\text{min}(\text{s})}$$ (20)

The normalized score ${\tilde{s}}_{i}$ quantifies the relative importance of the i−th residue. By aligning high-dimensional semantic embeddings with biologically grounded residue-level attributions, SHAP-Res provides a direct interpretability pathway, enabling meaningful insights into sequence–function relationships.

### Model construction and selection

This section presents a comprehensive overview of the HyperACP model construction process, beginning with the preparation of the raw dataset and detailing each subsequent key stage. Initially, the deployment and loading of the pretrained large language model (specifically, the ESMC-600M version) in a local environment are described, along with the comparative analysis and rationale for base model selection. The application of the ANBS algorithm is then highlighted, emphasizing its role in addressing data imbalance and optimizing the augmentation of minority class samples. Subsequently, the optimization procedures for the machine learning model are detailed, encompassing hyperparameter tuning, model performance evaluation, and specific strategies for performance enhancement. Through this systematic workflow, HyperACP attains both high efficiency and accuracy in anticancer peptide classification.

### Local deployment of pre-trained language models

In accordance with the ESM license agreement, the code and model weights for ESMC are available for use in both non-commercial and permissive commercial settings. The ESMC model weights have been open-sourced on the Hugging Face Hub, enabling users to either execute the code locally and instantiate the model via PyTorch, or download the weights from Hugging Face for local loading. The latter approach is recommended, as local model loading is generally faster and avoids potential failures due to network instability. Prior to deployment, it is essential to ensure that the Python and PyTorch environments are properly configured, CUDA is available, the required ESM packages are installed, and the model is set to evaluation mode. The pseudocode for local model loading is as follows:

**Algorithm 2. ESMC-Based Protein Feature Extraction.**

**Input:**

  **Pre-trained ESMC model** (M): *ESMC-600M or ESMC-300M*

  **Protein sequence** (S)

  **Computation device:** *CUDA (if available) or CPU*

**Output:**

  **Logits (L)**

  **Protein embeddings (E)**

1  **Initialize Environment:**

2   Set infrastructure provider flag

3   Select computation device:

   If CUDA is available, assign device←CUDA

   Else, assign device←CPU

4 **Load Pre-trained ESMC Model:**

5   Choose model: M←ESMC-600M or ESMC-300M

6   Load model weights: M ← M.from_pretrained()

7   Assign model to selected device: M ← M.to(device)

8   Set model to evaluation mode: M.eval()

9 **Prepare Protein Sequence:**

10   Define input sequence: S

   Convert sequence to **ESMProtein** format

11 **Encode Protein Sequence:**

12   Pass sequence through encoder: T ← M.encode(S)

   Obtain **protein tensor representation**

13 **Compute Logits and Embeddings:**

14  Define logits configuration:

   Enable **sequence-level logits, Enable embedding extraction**

15    Perform model inference:

    L,E ← M.logits(T,LogitsConfig)

16 **Return Results:** Output **logits:**L, Output **embeddings:**E

ESMC has released three versions with differing parameter scales: 300M, 600M, and 6B, where the numeric designation corresponds to the number of model parameters. In this study, the 650M-parameter version of ESMC was selected and deployed on a Windows 11 system equipped with an NVIDIA GeForce RTX 4090 graphics card, which provides 24 GB of VRAM—sufficient to accommodate the computational demands of the model. The length of the peptide sequences processed does not exceed 50 residues. Detailed model parameters for the ESM series are summarized in Table 3, allowing researchers to select the most appropriate version according to available hardware resources and dataset characteristics.

**Table 3:** Models of the ESM series and their parameters.

ESM.pretrained	Layers	Params	Embedding Dim
ESM-IF1	20	124M	512
ESM-1v	33	650M	1280
ESM-MSA-1b	12	100M	768
ESM1b	33	650M	1280
ESM1(670M)	34	670M	1280
ESM1(85M)	12	85M	768
ESM1(43M)	6	43M	768
ESM2(15B)	48	15B	5120
ESM2(3B)	36	3B	2560
ESM2(650M)	33	650M	1280
ESM2(150M)	30	150M	640
ESM2(35M)	12	35M	480
ESM2(8M)	6	8M	320
ESMC(3B)	80	3B	2560
ESMC(600M)	36	600M	1152
ESMC(300M)	30	300M	960

### Optimization and combination of base models

In selecting the optimal backbone model for HyperACP, we focused on recent protein language models, excluding the older ESM1 series to leverage more extensive and current training data. Considering the computational overhead, we also limited our candidates to models under one billion parameters. The head-to-head comparison of the resulting ESM2 and ESMC models is detailed in Table 4. While the ESM2 series shows performance gains with scale, the ESMC models demonstrate clear superiority across all metrics. Notably, ESMC-600M achieved the highest scores across the board and was consequently chosen for integration into the HyperACP framework.

**Table 4:** Results of ten-fold cross-validation for ESM models with different parameters.

Model	Accuracy	AUC	F1 Score	MCC	Sensitivity	Specificity
ESM2_t6_8M	0.969	0.995	0.969	0.939	0.987	0.951
ESM2_t12_35M	0.970	0.996	0.970	0.940	0.989	0.951
ESM2_t30_150M	0.973	0.998	0.974	0.946	0.990	0.956
ESM2_t33_650M	0.979	**0.999**	0.980	0.959	0.993	0.966
ESMC_300m	0.979	**0.999**	0.979	0.958	0.992	0.965
ESMC_600m	**0.982**	**0.999**	**0.985**	**0.965**	**0.994**	**0.971**

To ensure high classification accuracy, robust generalization, and satisfactory interpretability in anticancer peptide prediction, both traditional machine learning models and deep learning networks were considered. To establish a fair comparative baseline and isolate the impact of different feature representation strategies, all models were evaluated on the same dataset, which was balanced by the ANBS algorithm to contain 1,478 positive and 1,478 negative samples. The results of the ten-fold cross-validation experiments are summarized in Table 5.

**Table 5:** Results of ten-fold cross-validation for different base models.

Model	Accuracy	AUC	F1	MCC	Sensitivity	Specificity
MLP	0.528	0.551	0.565	-0.008	0.763	0.224
RNN	0.558	0.517	0.653	0	0.900	0.100
LSTM	0.533	0.516	0.509	0	0.700	0.300
GRU	0.499	0.498	0.429	0	0.600	0.400
CNN	0.464	0.515	0.222	0.004	0.263	0.741
Logistic Regression	0.862	0.943	0.860	0.725	0.846	0.878
SVM	0.874	0.950	0.874	0.749	0.873	0.875
Random Forest	**0.984**	**0.999**	**0.984**	**0.969**	0.984	**0.985**
KNN	0.773	0.910	0.815	0.613	**0.999**	0.547
Gradient Boosting	0.958	0.992	0.960	0.918	0.980	0.938
XGBoost	0.976	0.998	0.976	0.952	0.988	0.963
Decision Tree	0.873	0.873	0.878	0.748	0.913	0.832
HistGradientBoosting	0.983	**0.999**	0.983	0.966	0.990	0.976

The evaluation first revealed that all neural network models (MLP, RNN, LSTM, GRU, CNN) performed poorly, with accuracies of 0.464–0.558 and near-zero or negative MCC scores, indicating predictions were no better than random. The CNN model, for example, was heavily biased, trading high specificity (0.741) for very low sensitivity (0.263). By comparison, the traditional machine learning models were far more effective. Specifically, Random Forest achieves the highest overall accuracy (0.984), an AUC of 0.999, and an F1 Score of 0.984, while also exhibiting strong robustness in handling class imbalance, as evidenced by its MCC of 0.969 and specificity of 0.985. HistGradientBoosting attains a sensitivity of 0.990 and maintains comparably high levels of overall performance, with an AUC of 0.999 and metrics closely aligned with those of Random Forest. Gradient Boosting also displays remarkable generalization capacity, with an AUC of 0.992, an MCC of 0.918, and an F1 Score of 0.960. In contrast, models such as k-Nearest Neighbors (KNN), Logistic Regression, and Decision Tree perform relatively poorly, particularly in adapting to complex, nonlinear feature spaces. For instance, the specificity of KNN is only 0.547, substantially lower than that of other models, indicating marked limitations in distinguishing between positive and negative samples [38–40].

Based on a comprehensive consideration of performance and stability, this study ultimately adopts a soft voting ensemble strategy, integrating the ESMC-600M model with three traditional machine learning algorithms: Random Forest, Gradient Boosting, and HistGradientBoosting. By leveraging the strong sequence representation capabilities of ESMC-600M and the complementary decision patterns of the ensemble learners, this approach effectively mitigates the potential fluctuations associated with individual models through collaborative voting, while preserving the high predictive capacity of each base learner. As a result, the overall model achieves enhanced robustness and generalization, ensuring optimal performance across multiple evaluation metrics and providing a strong methodological foundation for the accurate identification of anticancer peptides.

## Results and discussion

### Comparison of sampling algorithms on all base models

**Fig 2:**
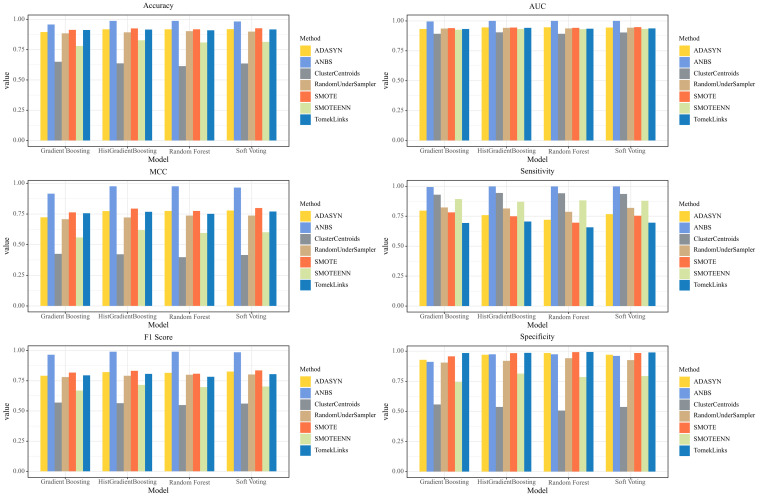
Comparative results of the ANBS algorithm and other sampling methods applied to base models.

To further validate the effectiveness of the ANBS sampling algorithm, a comparative analysis was conducted involving several commonly used sampling techniques, including ClusterCentroids, SMOTE, TomekLinks, SMOTEENN, ADASYN, and RandomUnderSampler. These algorithms were evaluated in conjunction with three base models as well as the proposed model using ten-fold cross-validation. The comparative results are presented in Fig 2.

Fig 2 provides a comprehensive comparison of various sampling strategies—including ANBS, ADASYN, SMOTE, SMOTEENN, ClusterCentroids, RandomUnderSampler, and TomekLinks—applied to multiple base models (Gradient Boosting, HistGradientBoosting, Random Forest, and Soft Voting) in the context of anticancer peptide classification. Performance was evaluated across six key metrics: Accuracy, AUC, F1 Score, MCC, Sensitivity, and Specificity.

The results clearly demonstrate that the combination of ANBS with the Soft Voting ensemble model consistently achieves superior performance across all evaluation metrics, exhibiting strong discriminative power and boundary generalization capability. Notably, ANBS significantly enhances both Accuracy and AUC for all base models, with the most pronounced improvements observed in the Random Forest and Soft Voting configurations, where performance approaches near-perfect levels. This indicates that ANBS effectively optimizes the class structure within the training data, thereby enabling robust model discrimination in the presence of complex, nonlinear boundaries. The Soft Voting ensemble further amplifies the benefits of ANBS by integrating the predictive strengths of multiple base classifiers, resulting in a balanced improvement of both accuracy and robustness.

In metrics specifically addressing class imbalance, such as F1 Score and MCC, ANBS yields more stable and substantial improvements compared to other sampling methods. When combined with Soft Voting, both F1 and MCC are significantly elevated, reflecting enhanced identification of the minority class while maintaining accuracy in the majority class. This is attributable to ANBS’s adaptive neighborhood selection, which focuses on generating high-quality, non-redundant synthetic samples near decision boundaries. Soft Voting leverages these improvements by aggregating probabilistic predictions from multiple models, further strengthening classification performance. For Sensitivity and Specificity, ANBS demonstrates superior global balancing capabilities, particularly within the Soft Voting model. This combination achieves higher recall for the positive class without compromising specificity, thus avoiding the typical trade-off between minority and majority class accuracy seen in other methods. These results underscore ANBS’s effectiveness in optimizing sample structure and highlight its suitability for highly imbalanced biomedical datasets.

In summary, the integration of ANBS—an adaptive, structure-aware sampling approach—with a Soft Voting ensemble yields a classification framework that is efficient, robust, and broadly applicable. This framework substantially improves the modeling of complex and imbalanced data distributions, making it particularly well-suited for biomedical applications such as anticancer peptide identification. The findings provide both theoretical support and practical guidance for future development of structurally optimized sampling and ensemble strategies.

### Performance analysis of ANBS algorithm

The original training set comprised 487 positive and 1,479 negative samples. With the ANBS algorithm’s target class ratio set to the default value of 1.0, the positive class was adaptively augmented to 1,478 samples, while the negative class was marginally reduced to 1,478 samples. These results demonstrate that ANBS can effectively enhance the representation of the minority class while preserving the structural integrity of the majority class, thereby mitigating the issue of class imbalance and providing a more balanced data foundation for subsequent classification models.

**Fig 3:**
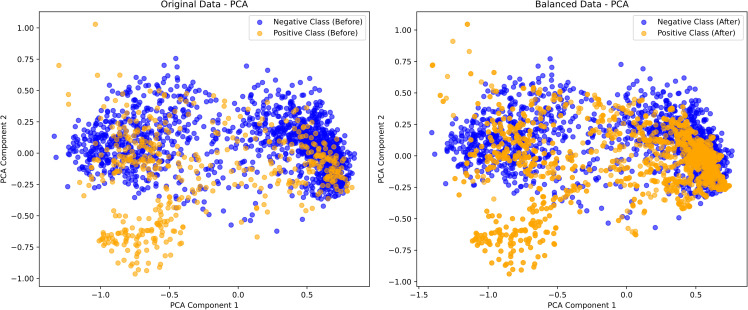
Visualization results of the ANBS algorithm (PCA).

**Fig 4:**
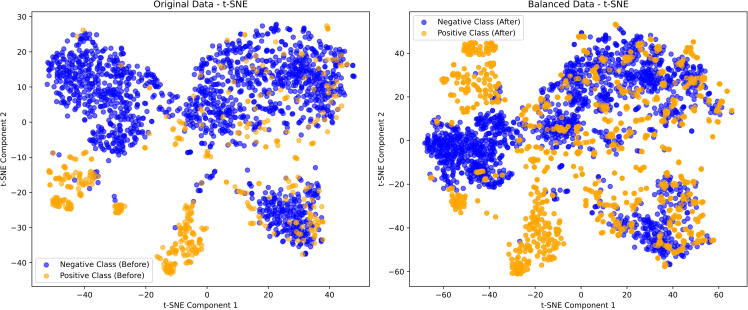
Visualization results of the ANBS algorithm (t-SNE).

To illustrate the impact of ANBS on imbalanced datasets, we employed Principal Component Analysis (PCA) and t-distributed Stochastic Neighbor Embedding (t-SNE) to compare the feature distributions before and after applying ANBS. The results, based on ESMC (600M), are presented in Figs 3 and 4.

As shown in Fig 3, the original feature space is highly dispersed, with indistinct boundaries between positive (orange) and negative (blue) samples. Positive samples are frequently enclosed by negative ones, resulting in low inter-class separability and a limited number of boundary samples. The absence of sufficient boundary information hinders machine learning models from accurately capturing critical class distinctions, thereby reducing their generalization ability and robustness. After applying the ANBS algorithm, the distributions of positive and negative samples become more balanced and separable. Positive samples form more compact clusters, while negative samples expand accordingly, particularly near the decision boundary. These results suggest that ANBS effectively mitigates clustering issues arising from class imbalance and improves inter-class separability.

Fig 4 illustrates the data distribution after dimensionality reduction using t-SNE. In the original imbalanced dataset, substantial overlap remains between positive and negative samples, particularly in regions with sparse positive instances where negative samples dominate the feature space. This overlap hinders the model’s ability to distinguish between classes during training. After rebalancing with ANBS, the positive and negative samples become more clearly separated, with each class forming compact and distinguishable clusters in the t-SNE projection. These findings indicate that ANBS adaptively augments the minority class, enhances class separation, and facilitates a clearer and more optimized feature representation for downstream classification tasks.

Both PCA and t-SNE visualizations indicate that the ANBS-balanced data exhibit significantly improved class separability, reduced sample overlap, and increased classification accuracy. By appropriately augmenting the minority (positive) class while preserving data diversity and minimizing redundancy, ANBS further enhances the generalization capability of the classification model. The improved class balance results in clearer decision boundaries, thereby facilitating more effective model training, particularly in scenarios involving class imbalance.

In this study, the ANBS (Adaptive Neighborhood-Based Sampling) algorithm demonstrates marked advantages in addressing class imbalance by targeting key limitations of traditional sampling methods. Unlike down-sampling, which risks losing structurally important samples, ANBS selectively removes majority class instances with weak neighborhood interaction to preserve essential data structure. Compared to up-sampling methods like SMOTE and ADASYN—which often generate redundant or invalid samples—ANBS integrates neighborhood selection with adaptive synthesis to create new minority class samples, primarily in boundary or sparse regions, thereby enhancing boundary representation and model discrimination.

The performance of ANBS, however, varies with the downstream classifier and dataset characteristics. In models sensitive to sample noise (e.g., KNN, decision trees), synthetic samples may disrupt local structure, while linear models (e.g., logistic regression) may not fully exploit improved boundary distributions. ANBS is particularly effective in settings with ambiguous boundaries and sparse minority regions—such as anticancer peptide classification—but yields limited gains when class boundaries are already well-defined.

Overall, ANBS is best suited for classifiers robust to noise and capable of modeling nonlinear relationships, such as Random Forest and Gradient Boosting. It offers a flexible and efficient approach to enhancing boundary information and improving minority class representation in complex biological datasets.

### Comparison of feature extraction methods

**Fig 5:**
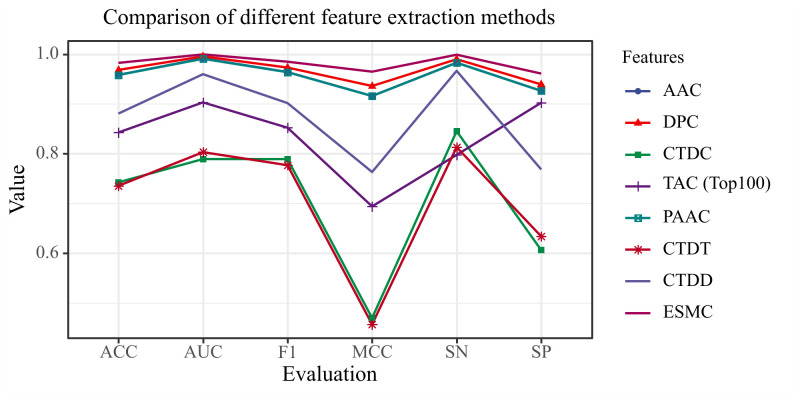
Comparison results between ESMC and other traditional feature extraction methods.

To systematically assess the performance of various feature extraction methods in anticancer peptide classification, this study compared seven widely used amino acid feature representations. These include frequency-based methods such as AAC, DPC, CTDC, PAAC, and CTDT; the distribution-based method CTDD; and the ranking-based method TAC (Top100). The initial dataset consisted of 487 positive and 1,479 negative samples. This was processed using the ANBS algorithm (with the default max_ratio = 1.0) to generate a balanced training set of 1,478 positive and 1,478 negative samples. To ensure a fair and rigorous comparison of the different feature extraction methods and to identify the optimal feature representation scheme, all evaluations were conducted under two fixed conditions. First, every method was benchmarked on the identical, ANBS-processed dataset (1,478 positive, 1,478 negative samples). Second, a fixed base model was used for all comparisons: an equally weighted soft-voting ensemble of Gradient Boosting, HistGradientBoosting, and Random Forest. Fig 5 presents the overall classification performance of these feature sets across six key metrics (Accuracy, AUC, F1 Score, MCC, Sensitivity, and Specificity), providing a clear basis for analyzing the impact of different feature representations on model outcomes.

The results indicate that ESMC-derived features consistently surpass all other methods across every evaluated metric, firmly establishing their superiority in amino acid sequence representation. Notably, ESMC features approach the optimal boundary in terms of Accuracy, AUC, F1 Score, and Specificity, reflecting exceptional performance in discriminative power, class separation, and generalization. ESMC also maintains a leading position in MCC and Sensitivity—two metrics that are particularly critical for evaluating robustness under class imbalance and the identification capability for the positive class (anticancer peptides). In contrast, traditional frequency-based or physicochemical property-based features (such as AAC, CTDC, and CTDT) offer marginal advantages in certain metrics but demonstrate limited overall performance. Furthermore, features like TAC (Top100) and CTDD exhibit substantial declines in MCC and Sensitivity, underscoring their limitations in boundary discrimination and positive class representation. These findings emphasize that conventional feature extraction methods, which typically rely on manually defined rules or low-level statistical descriptors, are insufficient for capturing the deep structural semantics and functional information intrinsic to protein sequences.

The deep semantic features extracted by the pretrained ESMC language model fundamentally address the limitations inherent in traditional feature extraction approaches. Through self-supervised learning on large-scale, unlabeled protein databases, ESMC autonomously learns complex and abstract sequence representations. This process enables the comprehensive capture of contextual dependencies among amino acids, long-range interactions, and latent structural information. Consequently, the resulting feature representations are more expressive and discriminative, providing a solid foundation for improved performance in downstream classification tasks.

In summary, the results presented in Fig 5 clearly demonstrate that ESMC-derived features consistently outperform traditional, manually designed features across all key metrics. Especially in the context of complex structures and sparse data—typical of anticancer peptide recognition—ESMC exhibits superior robustness and a heightened ability to capture biologically relevant information. This highlights the significant potential of pretrained language models as a promising direction for future protein representation methodologies.

### Comparison with existing methods

To systematically assess the robustness and generalization capacity of HyperACP under varying class distribution conditions, this study compared the performance of several state-of-the-art anticancer peptide classification methods on two independent test sets: ACP135 and ACP99. Notably, ACP135 is characterized by a pronounced class imbalance, with a positive-to-negative sample ratio of 1:2.7, which presents a considerable challenge for boundary discrimination and minority class detection. In contrast, ACP99 exhibits a more balanced class ratio of 1:1.59, offering a more representative evaluation of model generalization in practical scenarios. The comparative results of HyperACP on these two independent test sets are summarized in Tables 6 and 7.

**Table 6:** Performance of ensemble models on ACP135.

Methods	SN(%)	SP(%)	ACC(%)	MCC	AUC
OURs	89.15	92.22	**91.11**	**0.783**	**0.964**
ACP-ML	**93.87**	82.96	90.89	0.770	0.937
mACPred	87.95	83.81	87.07	0.659	0.899
iDACP	86.06	**97.47**	87.88	0.687	0.926
ACPred	91.40	71.92	85.66	0.648	0.911
ACP-MHCNN	78.35	33.85	59.02	0.136	N.A
ACPred-BMF	83.79	42.93	66.87	0.296	N.A

N.A.: non-applicable.

**Table 7:** Performance of ensemble models on ACP 99.

Methods	SN(%)	SP(%)	ACC(%)	MCC	AUC
OURs	**96.91**	**96.39**	**96.65**	**0.933**	**0.994**
ACP-ML	93.67	90.82	92.58	0.843	0.975
mACPred	88.24	91.86	89.45	0.777	0.953
ACPred	89.61	81.37	86.38	0.714	0.934
ACP-MHCNN	73.81	62.50	69.92	0.354	N.A
ACPred-BMF	92.04	62.94	75.78	0.561	N.A

N.A.: non-applicable.

Across both independent test sets, HyperACP demonstrates consistently strong performance on all six key evaluation metrics, particularly achieving the most balanced trade-off between SN and SP. On ACP135, although ACP-ML attains a higher sensitivity (93.87%) compared to HyperACP (89.15%), its specificity is considerably lower (82.96%), indicating a heightened risk of false positives due to compromised negative class accuracy. In contrast, HyperACP maintains high sensitivity while achieving a specificity of 92.22%, effectively reducing false positive rates without sacrificing the identification of positive samples. This reflects robust discriminative power in complex boundary scenarios.

This advantage is further accentuated in the more balanced ACP99 dataset, where HyperACP achieves both high sensitivity (96.91%) and specificity (96.39%), outperforming all other models by a significant margin. Moreover, HyperACP leads in critical overall performance and boundary quality metrics, with an MCC of 0.933 and an AUC of 0.994. In comparison, certain methods such as ACP-BMF or ACP-MHCNN perform adequately in some metrics but display markedly lower MCC values (e.g., ACP-BMF achieves only 0.296 in ACP135), highlighting limitations in boundary modeling and overall stability under varying class distributions.

Notably, in the highly imbalanced ACP135 dataset, some models—while exhibiting elevated sensitivity (e.g., ACPred with 91.4%)—suffer from low specificity (71.9%), resulting in a high false positive rate. In biomedical applications, this not only undermines practical utility but may also lead to misleading biological conclusions. Thus, the ability to balance sensitivity and specificity is of particular importance.

In summary, HyperACP exhibits robust boundary learning, effective class discrimination, and high generalizability across different class distribution scenarios. These strengths are attributed to the integration of ESMC-derived deep protein representations, the ANBS adaptive boundary sampling strategy, and a soft voting ensemble of multiple base models. Collectively, these components enable HyperACP to surpass existing methods in both generalization performance and real-world applicability, particularly in challenging, biologically complex, and imbalanced classification tasks.

### Ablation study

**Fig 6:**
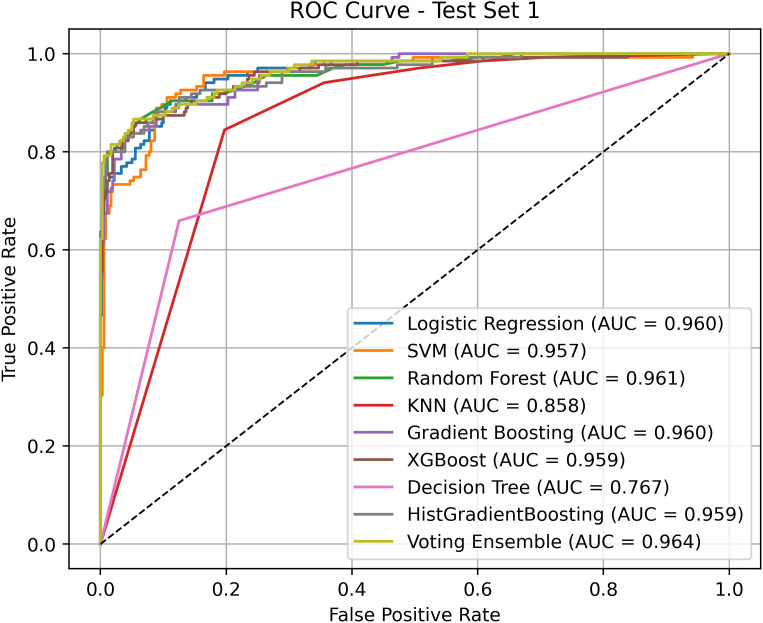
ROC curve comparison results between HyperACP and other models on ACP135.

**Fig 7:**
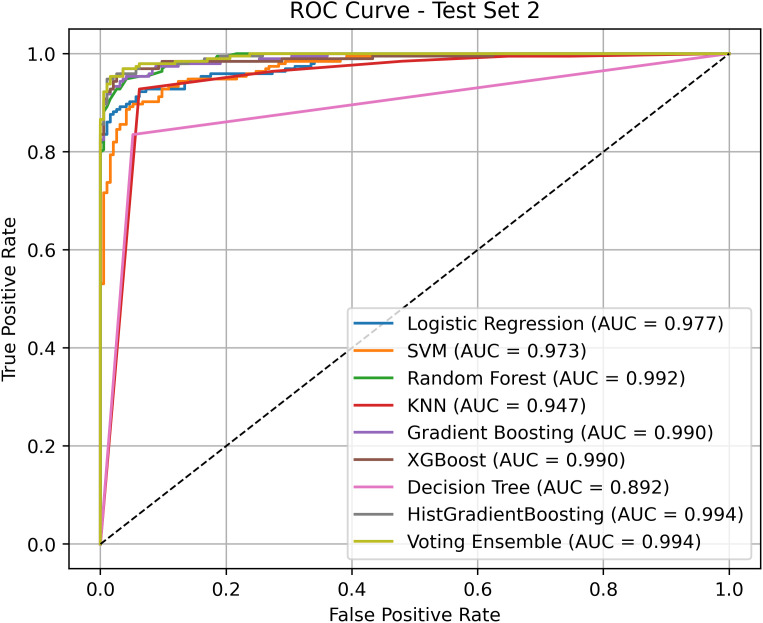
ROC curve comparison results between HyperACP and other models on ACP99.

To further evaluate the effectiveness of the proposed ensemble model in anticancer peptide classification, a series of ablation experiments were conducted to assess the independent performance of various base classifiers under identical feature inputs and a unified sampling strategy. These experiments also serve to verify the ensemble benefits of the Voting Ensemble approach. Notably, all models in this study were trained following the application of the ANBS (Adaptive Neighborhood-Based Sampling) algorithm, which has been previously shown to significantly improve boundary detection and class balance. Figs 6 and 7 present the ROC curves and corresponding AUC values for each model on the two independent test sets (ACP135 and ACP99), thereby reflecting their true discriminative capabilities under consistent data processing conditions.

On Test Set 1, individual models such as Random Forest (AUC = 0.961), Gradient Boosting (AUC = 0.960), and Logistic Regression (AUC = 0.960) demonstrated strong predictive performance. However, the Voting Ensemble further integrated these base models, resulting in a higher overall ROC curve, a noticeably reduced false positive rate, and a smoother, more optimal ROC profile. In contrast, models such as KNN and Decision Tree, despite the support of ANBS, remained constrained by limited structural expressiveness, achieving lower AUC values of 0.858 and 0.767, respectively. These results suggest that more complex models are better able to capitalize on the structural enhancements provided by ANBS.

On Test Set 2, all models exhibited improved AUC values, demonstrating the strong transferability of the data generated by ANBS across different test sets. Random Forest and HistGradientBoosting achieved AUC of 0.992 and 0.994, respectively, indicating robust performance. The Voting Ensemble attained the highest AUC (0.994), matching that of HistGradientBoosting, thereby further validating its capacity to effectively integrate multiple strong base learners. Notably, the ROC curve of the Voting Ensemble is smoother and maintains a steep rise in the high true positive rate (TPR) region, suggesting that it effectively suppresses false positives while sustaining a high detection rate.

In summary, the ablation experiments confirm that, given a unified and structurally optimized dataset, the Voting Ensemble achieves more stable and superior generalization performance by integrating the decision boundaries of multiple models. This underscores its practical value as the final classifier in complex and imbalanced anticancer peptide classification tasks. Moreover, the experiments highlight the essential role of ANBS in enhancing data quality, serving as a critical foundation for the overall performance improvements observed.

### Interpretability analysis work—Analysis of amino acids and their physicochemical properties

**Fig 8:**
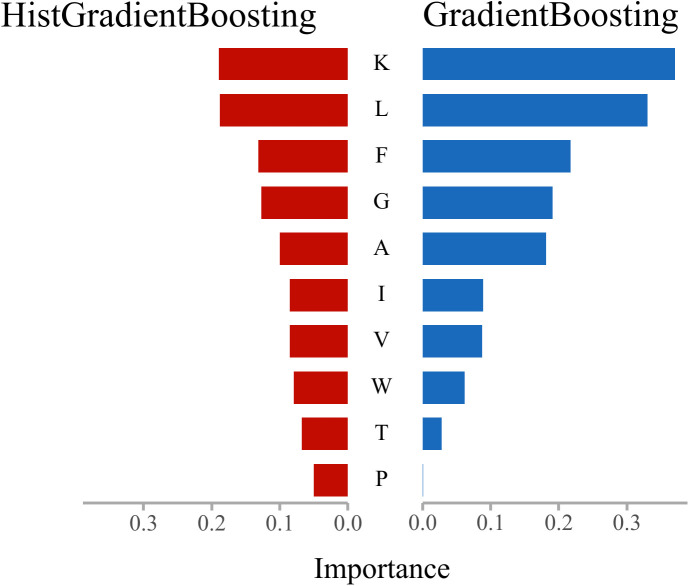
Results of feature importance comparison between HistGradientBoosting and GradientBoosting.

**Fig 9:**
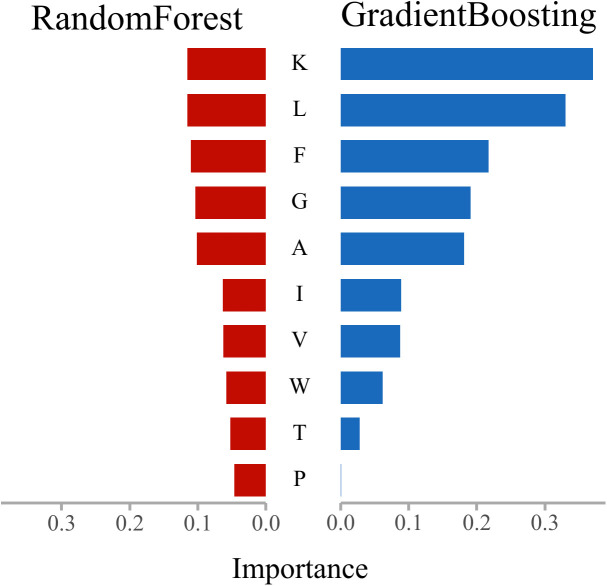
Results of feature importance comparison between RandomForest and GradientBoosting.

**Fig 10:**
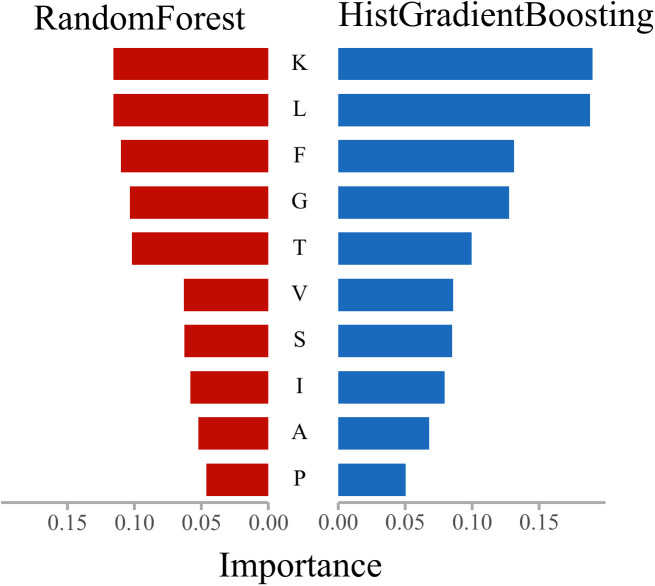
Results of feature importance comparison between RandomForest and HistGradientBoosting.

In this section, feature importance rankings for three mainstream ensemble models—Random Forest, Gradient Boosting, and HistGradientBoosting—were analyzed using the SHAP-Res algorithm applied to amino acid frequency features. After normalization, the results were examined to elucidate the feature attention mechanisms underlying anticancer peptide recognition, thereby enhancing model interpretability. The analysis further integrates the physicochemical properties of amino acids [25,26] to interpret model findings from a biological perspective. Through pairwise comparisons, common and distinct residue preferences among the models were identified, revealing the biological rationale underlying their discriminative mechanisms. The comparative results are presented in Figs 8–10.

As shown in Fig 8, the two gradient boosting models—HistGradientBoosting and GradientBoosting—exhibit a high degree of consistency in their feature attention patterns, consistently ranking K (Lysine), L (Leucine), F (Phenylalanine), and G (Glycine) as the most influential residues. This suggests that both models preferentially identify residues associated with hydrophobicity (L, F), basicity (K), and flexibility (G) during the learning process. These amino acids are frequently implicated in membrane binding, structural bending, or the formation of charged regions within functional peptides, and are closely linked to properties such as membrane permeability and cytotoxicity in anticancer peptides. Minor differences are observed in the ranking of long-tail features: HistGradientBoosting assigns greater importance to neutral or polar residues such as Isoleucine (I), Tryptophan (W), and Threonine (T), whereas GradientBoosting focuses more on the top-ranked residues. The histogram-based optimization employed by HistGradientBoosting may enhance its sensitivity to local frequency variations and sparsely distributed signals, thereby broadening its attention to structurally perturbative residues.

As illustrated in Fig 9, although both Random Forest and Gradient Boosting are ensemble models, their underlying mechanisms differ—Random Forest employs weighted residual iteration, while Gradient Boosting is based on majority voting. Despite these methodological differences, both models display complete agreement in their top five most important residues (K, L, F, G, A), indicating that these amino acids provide strong classification signals, with particular emphasis on the high and consistent importance assigned to K (Lysine), known for its strong basicity and high hydrophilicity. This consensus underscores K as a pivotal residue for positive class discrimination. Notably, Gradient Boosting concentrates its feature importance on the leading residues (e.g., assigning a weight of 0.35 to K), whereas Random Forest distributes feature importance more evenly across the top features. This suggests that Random Forest prioritizes overall distributional balance, while Gradient Boosting accentuates the most prominent discriminative features. Such differences in feature attention may partly account for the marginally superior performance and boundary discrimination observed with Gradient Boosting.

As shown in Fig 10, Random Forest and HistGradientBoosting exhibit similar overall feature ranking structures; however, two notable distinctions emerge. HistGradientBoosting assigns greater importance to small polar residues such as Glycine (G), Threonine (T), and Serine (S), which are often associated with peptide chain flexibility, conformational dynamics, and short-range interactions. This suggests that HistGradientBoosting may be more attuned to local structural instability and subtle variations within protein sequences. In contrast, Random Forest continues to place greater emphasis on dominant features such as Lysine (K), Leucine (L), and Phenylalanine (F), indicating a stronger reliance on globally stable classification signals and reduced sensitivity to minor variations among weaker boundary features. This comparison highlights the tendency of Random Forest to prioritize broadly stable attributes in model integration, while HistGradientBoosting more actively fine-tunes boundaries and leverages nuanced distributional signals. These observations are further supported by the ROC and AUC performance results discussed previously.

The comparative analysis demonstrates that residues such as Lysine (K), Leucine (L), Phenylalanine (F), and Glycine (G) consistently receive high importance across all models, identifying them as core features for anticancer peptide discrimination. This finding aligns with prior research—for example, Ratasark Summart et al. (2025) reported that peptides enriched in hydrophobic residues (e.g., L, F) and positively charged residues (e.g., K, R) exhibit increased membrane insertion and cytolytic activity, thereby contributing to anticancer effects [41]. Furthermore, HistGradientBoosting displays heightened sensitivity to structural-perturbing residues, such as small polar or aromatic amino acids (e.g., T, S, W), indicating a stronger response to protein structural flexibility. Literature supports the significant role of flexible peptides in membrane penetration and apoptosis induction, corroborating the model’s interpretability findings [42]. Gradient Boosting excels at amplifying dominant features and highlighting key residues with strong classification impact, while Random Forest distributes feature importance more evenly, which may confer robustness in tasks with imbalanced class distributions. These distinctions in model learning mechanisms have been noted in previous studies and are evident in the present analysis of ROC performance and feature response profiles [29,43,44].

Notably, the models’ recognition preferences for specific amino acids are closely associated with physicochemical properties such as hydrophobicity, charge, and flexibility, reinforcing the notion that sequence-level statistical information can partially capture biological function [45]. This underscores the value of synergistically optimizing feature engineering and model architecture, and supports the integration of pretrained representations (e.g., ESMC) with structure-aware sampling strategies (e.g., ANBS).

In summary, interpretability analysis of the three models elucidates the critical roles of key amino acid residues in anticancer peptide recognition, highlights differences in feature modeling among various learning structures, and provides a robust foundation for the development of predictive models with both biological plausibility and algorithmic reliability.

## Conclusion

ACPs hold significant promise as next-generation therapeutics due to their high selectivity, low toxicity, and ability to overcome multidrug resistance—advantages that make them compelling alternatives to conventional chemotherapeutic agents. In this work, we present HyperACP, a comprehensive framework for anticancer peptide prediction that integrates three key components—deep semantic encoding with ESMC, boundary-aware resampling via ANBS, and residue-level interpretation through SHAP-Res. Empirical evaluations on multiple imbalanced datasets demonstrate that HyperACP consistently outperforms state-of-the-art baselines across ACC, MCC, AUC, and F1, confirming its robustness and superior generalization. Moreover, SHAP-Res reveals biologically meaningful residue attributions (e.g., K, L, F, G), validating the plausibility of our predictions. Overall, HyperACP not only advances the predictive performance for ACP discovery but also provides transparent mechanistic insights, paving the way for future functional peptide research and precision drug development.

Despite the effectiveness of our proposed framework, several limitations remain. First, our framework is currently tailored for anticancer peptide classification, and its generalizability to other biological sequence types (e.g., enzymes or transporters) remains to be validated. Second, the ANBS sampling strategy operates in the Euclidean space of embeddings, which may inadequately reflect the true semantic geometry, especially in highly non-linear manifolds. Third, SHAP-Res relies on SHAP attributions derived from ensemble classifiers, whose additive assumptions may fail to capture complex residue interactions. Lastly, the ensemble training and residue-level explanation introduce non-trivial computational overhead. Future work will explore more expressive geometric sampling, task-specific embedding adaptation, and efficient attribution methods to address these challenges.
